# Supplementary material for: Graph Attention Site Prediction (GrASP): Identifying Druggable Binding Sites Using Graph Neural Networks with Attention
Source: bioRxiv. 2023 Jul 28:2023.07.25.550565. Preprint. [Version 1] doi: 10.1101/2023.07.25.550565 (PMC10402091; doi:10.1101/2023.07.25.550565)
Supplement: Supplement 1 [file media-1.pdf]

Supporting Information for "Graph Attention Site Prediction (GrASP): Identifying Druggable Binding Sites Using Graph Neural Networks with Attention"

Zachary Smith,[1,2,a] Michael Strobel,[3,a] Bodhi P. Vani,[1] and Pratyush Tiwary[1,4,b]

[1] *Institute for Physical Science and Technology, University of Maryland, College Park 20742, USA.*

[2] *Biophysics Program, University of Maryland, College Park 20742, USA.*

[3] *Department of Computer Science, University of Maryland, College Park 20742, USA.*

[4] *Department of Chemistry and Biochemistry, University of Maryland, College Park 20742, USA.*

[a] These authors contributed equally.

[b] Electronic mail: ptiwary@umd.edu

Atom-Scale Features
Atom Type
Local Density of Atoms (9 features, density within spheres ranging from 2 - 10 Å)
Solvent Accessible Surface Area
Formal Charge
Number of Bonds with Heavy Atom
Ring Membership
Aromaticity
Mass
Hybridization
Hydrogen Bond Donor/Acceptor
Hydrophobicity
**Amino Acid-Scale Features**
Residue Name
Residue Polarity
Acidity (Acidic/Basic/Neutral)
Charge (Positive/Negative/Neutral)
**Edge Features**
Inverse Distance
Bond Order (including unbonded)

TABLE S1: GrASP input features.

I. SC-PDB DATASET PREPARATION

Unlabeled ligands in the modified sc-PDB dataset were identified by matching their chemical composition to labeled ligands from an entry with the same PDB ID. This criteria was chosen to avoid adding ligands not present in the original sc-PDB in order to be consistent with the sc-PDB's requirement that ligands must be biologically relevant. The count of each non-hydrogen element in the ligand was compared to the labeled ligands and matches were recorded. The relatively loose criteria of element count was chosen to avoid false negatives due to inconsistencies in bond perception and ligands with a different ordering of elements,

different sybyl atom types, or different residue names were labeled for visual inspection to confirm they were not identified as duplicates in error.

Unlabeled ligands were identified as buried using the ratio of solvent accessible surface area (SASA) in the protein complex to SASA in vacuum. Ligands were classified as buried if they were either below 30% solvent accessible or if their fraction accessible was no more than 10% above their labeled counterpart's fraction. These criteria use a conservative definition of buried ligands with 30% accessible surface chosen because 95% of the labeled ligands fall below this threshold. The second part of the criteria accounts for ligands that have over 30% surface exposure in their binding modes. We found that ligands with long tails may be up to 60% accessible in their labeled binding mode and this comparison to the labeled ligands identifies the small number of cases where unlabeled ligands are symmetric to their labeled counterparts but over 30% accessible. The use of two criteria allows the base threshold of 30% to be low enough to avoid false positives while comparison to the labeled fraction accessible catches false negatives that would arise due to unique binding modes.

II. BINDING SITE LABELS

We train GrASP using continuously valued binding site labels with the following sigmoid form where y_i is the class label for protein atom i and d_i is the distance from protein atom i to the nearest ligand heavy atom.

$$y_i = \mathrm{Sigmoid}(-3(d_i - 5)) \tag{1}$$

This can be viewed as a smoothed version of a 5 Å binding site definition where the labels decrease from 1 to 0 in roughly the region between 4 - 6 Å as opposed to a discrete boundary at 5 Å. Both the midpoint and slope of this sigmoid were tuned as hyperparameters to optimize the top N DCA recall on the validation set.

III. CONVEX HULL CENTER CALCULATION

To calculate the center of a convex hull, the hull is treated as a solid object with uniform density and its center of mass is calculated. This is accomplished by breaking the hull into tetrahedrons and taking the volume-weighted average of these tetrahedrons' centroids.

IV. SEMANTIC SEGMENTATION METRICS

We use the following metrics to evaluate the performance of semantic segmentation:

- Area under the receiver operating characteristic curve (ROC AUC): A classification threshold invariant metric that measures the trade-off between the true positive rate and false positive rate as the classification threshold is varied. Here we use macro averaging to give equal weight to each category instead of each sample because there is heavy class imbalance between site and non-site atoms.
- Area under the precision-recall curve (PR AUC): A classification threshold invariant metric that measures the trade-off between precision and recall as the classification threshold is varied. Similar to ROC AUC but less sensitive to class imbalance.
- Matthews correlation coefficient[1] (MCC): A correlation metric commonly used for tasks where there is class imbalance. A value of 1 means all predictions are correct while a value of -1 means all predictions are incorrect and a value of 0 corresponds to random predictions. The MCC is shown in Eq. 2 where T, F, P, and N correspond to true, false, positive, and negative respectively.

$$MCC = \frac{TP \times TN - FP \times FN}{\sqrt{(TP + FP)(TP + FN)(TN + FP)(TN + FN)}} \tag{2}$$

COACH420(Mlig+)			HOLO4K(Mlig+)		
ROC AUC	PR AUC	MCC	ROC AUC	PR AUC	MCC
.97	.75	.64	.98	.79	.70

TABLE S2: GrASP atom-wise semantic segmentation metrics on COACH420(Mlig+) and HOLO4K(Mlig+).

REFERENCES

[1] Matthews, B. Comparison of the predicted and observed secondary structure of T4 phage lysozyme. Biochimica et Biophysica Acta (BBA) - Protein Structure **1975**, 405, 442–451.
